# Dual Antiplatelet Therapy in Patients Aged 75 Years and Older with Coronary Artery Disease: A Meta-Analysis and Systematic Review

**DOI:** 10.1155/2022/3111840

**Published:** 2022-09-15

**Authors:** Garly Saint Croix, Spencer C. Lacy, Amre Gazzhal, Michel Ibrahim, Medeona Gjergjindreaj, Jorge Perez, Malik Shehadeh, Karthik Vedantam, Christian Torres, Nirat Beohar, Esteban Escolar

**Affiliations:** ^1^Columbia University Division of Cardiology at Mount Sinai Medical Center, Miami Beach, Florida, USA; ^2^Loyola University Medical Center, Maywood, Illinois, USA; ^3^Temple University Medical Center, Philadelphia, Pennsylvania, USA

## Abstract

**Objectives:**

This systematic review and meta-analysis evaluates the safety and efficacy of dual antiplatelet therapy (DAPT) in elderly patients with acute coronary syndrome (ACS).

**Background:**

The safety and efficacy of DAPT in elderly patients with ACS is not well characterized.

**Methods:**

We performed a systematic literature review to identify clinical studies that reported safety and efficacy outcomes after DAPT for ACS in elderly patients. The primary outcomes of primary efficacy endpoint rates and bleeding event rates were reported as random effects risk ratio (RR) with 95% confidence interval. No prior ethical approval was required since all data are public.

**Results:**

Our search yielded 660 potential studies. We included 8 studies reporting on 29,217 patients. There was a higher risk of bleeding event rates in elderly patients treated with prasugrel or ticagrelor when compared to clopidogrel with a risk ratio of 1.17 (95% CI 1.08 to 1.27, *p* < 0.05). There was no difference in primary efficacy endpoint rates between elderly patients treated with prasugrel or ticagrelor when compared to clopidogrel with a risk ratio of 0.85 (95% CI 0.68 to 1.07, *p*=0.17).

**Conclusions:**

This systematic review and meta-analysis suggests that DAPT with prasugrel or ticagrelor compared to clopidogrel is associated with a higher risk of bleeding events in elderly patients with ACS. There was no difference in the primary efficacy endpoints between the two treatment groups.

## 1. Introduction

Dual oral antiplatelet therapy (DAPT) with aspirin and P2Y12 receptor inhibitors has had a key role in the management of patients with acute coronary syndrome (ACS) and remains the treatment of choice to prevent in-stent thrombosis [1]. In patients with ACS undergoing percutaneous coronary intervention (PCI), a loading dose of DAPT (either aspirin + clopidogrel or aspirin + ticagrelor) is recommended as early as possible by the latest ESC guidelines [2]. Although current guidelines recommend the new and more predictable P2Y12 receptor inhibitors ticagrelor and prasugrel in ACS patients given their superiority to clopidogrel in preventing major adverse cardiovascular events (MACE), they have been associated with higher risk of bleeding especially in elderly patients [3].

Elderly patients contribute to a large proportion of patients with ACS and have often been underrepresented in the randomized trials that provided evidence for guidelines [4]. For example, in the TRITON TIMI 38 (Trial to Assess Improvement in Therapeutic Outcomes by Optimizing Platelet Inhibition with Prasugrel-Thrombolysis in Myocardial Infarction 38) and PLATO (Platelet Inhibition and Patient Outcomes) trials, elderly patients (age >75 years) accounted for only 13% and 15% of the study populations, respectively [5, 6]. Elderly patients are more susceptible to the adverse effects of DAPT with bleeding being one of the most common complications associated with prolonged hospitalization and increased mortality. However, investigations about the safety and efficacy of DAPT in this group are scarce [7].

In this meta-analysis and systematic review, we evaluate the impact of DAPT on clinical and bleeding outcomes in elderly patients with ACS.

## 2. Methods

The main objective of this review was to assess the safety and efficacy of ticagrelor or prasugrel compared to clopidogrel in elderly patients with ACS. We used the Preferred Reporting Items for Systematic Reviews and Meta-Analyses (PRISMA) statement extension for network meta-analysis. The PRISMA flow diagram was used to depict the four phases of the review including identification, screening, eligibility, and inclusion. The PRISMA statement contains a checklist of items required of systematic reviews and meta-analyses. The review was not registered a priori. No ethical approval was required since this meta-analysis uses only public published data.

### 2.1. Search Strategy

We performed a systematic literature review to identify randomized and non-randomized clinical studies that reported the use of DAPT in elderly patients with ACS. Searches were limited to peer-reviewed primary research articles published in English up to December 1^st^, 2021. This research involved human subjects and described the clinical impact of DAPT in elderly patients with ACS. We developed the search strategy according to available guidance from the Cochrane Collaboration.

The search strategy in PubMed explored Medical Subject Heading (MeSH) terms related to elderly patients with ACS treated with DAPT. The articles found to be relevant during the search were stored in EndNote. Selected articles underwent full evaluation to assess their potential inclusion in the systematic review.

### 2.2. Study Selection

Articles were selected for inclusion based on predefined criteria, which included age, sex, DAPT, bleeding, MACE events, and the primary or secondary outcomes being mortality, bleeding, and efficacy outcomes. Exclusion criteria were patients with elective PCI without ACS. We excluded case reports and studies with fewer than 10 subjects.

Two authors (GS and SL) independently read the trials and screened the abstracts to choose potentially relevant articles. Selected articles underwent full evaluation to assess their potential inclusion in the systematic review.

### 2.3. Definition of Elderly Patients

Each study defined elderly patients based on an arbitrary age. An age greater than 75 years old was defined as elderly in 5 of the included studies. Ages greater than 65, 70, and 80 were each used as cutoffs by 3 of the included studies.

### 2.4. Definition of Outcomes

Primary efficacy outcomes were defined separately by each included study. Most studies used a composite of death, myocardial infarction, or stroke during the follow-up period.

Bleeding events were defined separately by each included study. Most studies used TIMI major or minor bleeding or PLATO major or minor bleeding, as previously defined [8, 9].

### 2.5. Risk of Bias

The risk of bias was assessed using the Cochrane tool for assessing the risk of bias in randomized controlled trials (RCTs) [10]. The risk of bias was assessed by two independent reviewers (GS and SL).

### 2.6. Statistical Analysis

Data were analyzed using Review Manager Software 5.4. We used fixed effects to assess the combined risk estimates according to I2 statistics. Analysis to determine sensitivity and publication bias was detected by funnel plots. *p* < 0.05 was considered statistically significant.

## 3. Results

### 3.1. Literature Search

**Figure 1:**
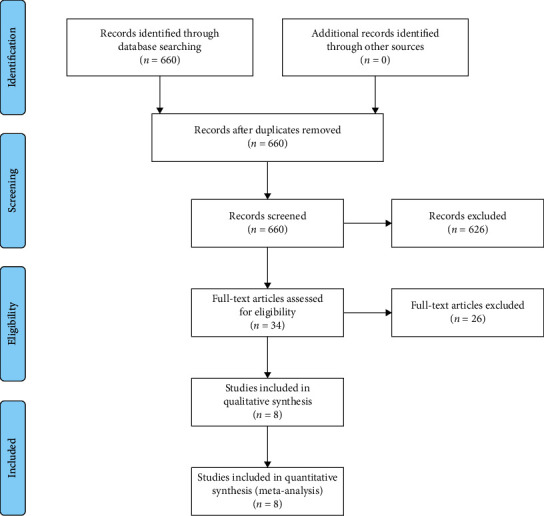
Flowchart of the included studies.

Our search yielded 660 potential studies. We excluded 626 studies at the abstract level and selected 34 full-text articles for detailed assessment; 8 studies were ultimately included in our systematic review and meta-analysis.  Figure 1 describes the flowchart of included studies.

### 3.2. Baseline Characteristics of the Studies

Table 1 shows the baseline characteristics of the included studies. All studies were published between 2007 and 2020. The 8 studies reported on 29,217 patients. Several of the included studies did not provide demographic data stratified by age, so the comparison of baseline characteristics in our target population of elderly patients is limited.

### 3.3. Risk of Bias

The risk of bias revealed adequate randomization, allocation concealment, and blinding in the 6 RCTs included in this study. The 2 non-RCTs included in this study were registry analyses that had appropriate selection and ascertainment approaches, while confounding adjustments were limited due to the observational design. Overall, the risk of bias for clinical outcomes was low in the RCTs and high in the non-RCTs.

### 3.4. Primary Efficacy Outcomes and Bleeding Events in Elderly Patients with CAD

**Figure 2:**
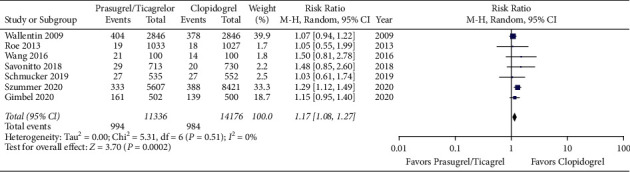
Forest plot of bleeding event rates for prasugrel or ticagrelor versus clopidogrel in elderly patients with acute coronary syndrome (CI = confidence interval).

**Figure 3:**
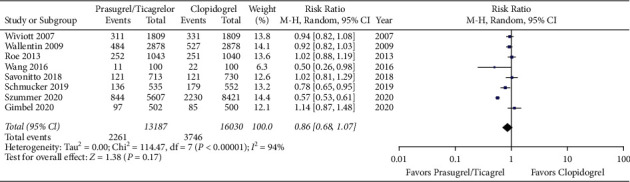
Forest plot of primary efficacy endpoint rates for prasugrel or ticagrelor versus clopidogrel in elderly patients with acute coronary syndrome (CI = confidence interval).

**Figure 4:**
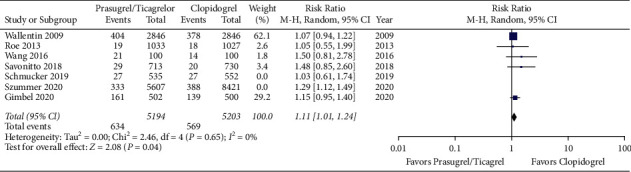
Forest plot of bleeding event rates for prasugrel or ticagrelor versus clopidogrel in elderly patients with acute coronary syndrome. The 2 non-randomized clinical trials by Schmucker et al. and Szummer et al. are excluded (CI = confidence interval).

**Figure 5:**
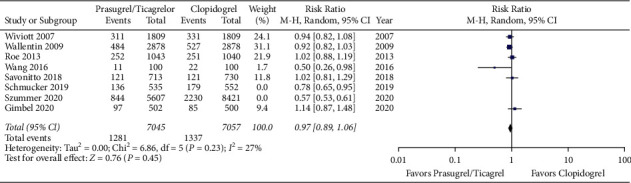
Forest plot of bleeding event rates for prasugrel or ticagrelor versus clopidogrel in elderly patients with acute coronary syndrome. The 2 non-randomized clinical trials by Schmucker et al. and Szummer et al. are excluded (CI = confidence interval).

Meta-analysis of the included studies revealed a higher risk of bleeding event rates in elderly patients treated with prasugrel or ticagrelor when compared to clopidogrel with a risk ratio of 1.17 (95% CI 1.08 to 1.27, *p* < 0.05). The forest plot for this comparison is shown in Figure 2. There was no difference in primary efficacy endpoint rates between elderly patients treated with prasugrel or ticagrelor when compared to clopidogrel with a risk ratio of 0.85 (95% CI 0.68 to 1.07, *p*=0.17). The forest plot for this comparison is shown in Figure 3. Meta-analysis with the non-RCT excluded revealed similar results as shown in Figures 4 and 5. The statistical heterogeneity for bleeding events was low with an I^2^ value of 0%. The statistical heterogeneity for primary efficacy endpoints was high with an I^2^ value of 94%.

## 4. Discussion

This systematic review and meta-analysis suggests that DAPT with prasugrel or ticagrelor compared to clopidogrel is associated with a higher risk of bleeding events in elderly patients with ACS. Our findings are derived from 8 studies reporting clinical outcomes in 29,217 patients [5, 9, 11–16]. There was no difference in the primary efficacy endpoints in DAPT with prasugrel or ticagrelor compared to clopidogrel in this patient population.

These findings provide a better understanding of the overall safety of DAPT in elderly patients assessed in various clinical trials. The CURE (Clopidogrel in Unstable Angina to Prevent Recurrent Events) trial demonstrated that clopidogrel was more effective than placebo in patients with ACS at the cost of increased risk of major bleeding regardless of age [17]. The benefit of prasugrel therapy compared to the risk of bleeding in elderly patients with ACS was shown to have a neutral net clinical benefit in the TRITON-TIMI 38 (Trial to Assess Improvement in Therapeutic Outcomes by Optimizing Platelet Inhibition with Prasugrel-Thrombolysis in Myocardial Infarction 38) trial [5]. Low-dose prasugrel and clopidogrel were shown to have similar efficacy and bleeding outcomes in elderly patients in the TRILOGY ACS (Targeted Platelet Inhibition to Clarify the Optimal Strategy to Medically Manage Acute Coronary Syndromes) study [11]. In the ELDERLY ACS II (Elderly Acute Coronary Syndrome 2) trial, low-dose prasugrel and clopidogrel showed similar primary endpoints in elderly patients with ACS [13]. In a substudy of elderly patients in the PLATO (Platelet Inhibition and Patient Outcomes) trial, there was no significant difference in major bleeding events between patients treated with ticagrelor versus clopidogrel [6]. In a more recent analysis, clopidogrel was shown to have decreased bleeding events with similar efficacy rates when compared to ticagrelor in elderly patients with ACS in the POPular AGE (Ticagrelor or Prasugrel Versus Clopidogrel in Elderly Patients With an Acute Coronary Syndrome and a High Bleeding Risk: Optimization of Antiplatelet Treatment in High-Risk Elderly) trial [16]. Ticagrelor was shown to decrease major ischemic events without increasing bleeding events in elderly patient with STEMI in the Bremen STEMI registry [14]. However, in the SWEDEHEART (Swedish Web System for Enhancement and Development of Evidence-Based Care in Heart Disease Evaluated According to Recommended Therapies) trial, ticagrelor was associated with a higher risk of bleeding and death when compared to clopidogrel in elderly patients with MI [15].

Additional studies have evaluated DAPT in elderly patients for extended periods of time over 1 year after the index event. DAPT extended for 30 months versus aspirin only was associated with decreased ischemic events and stent thrombosis at the expense of increased bleeding events in the DAPT (Dual Antiplatelet Therapy) trial. However, the benefit of prolonged DAPT was decreased and the bleeding event rates increased when stratified by age [18]. In the PEGASUS-TIMI 54 (Prevention of Cardiovascular Events in Patients With Prior Heart Attack Using Ticagrelor Compared to Placebo on a Background of Aspirin-Thrombolysis In Myocardial Infarction 54) trial, ticagrelor and aspirin were shown to have a benefit on the 3-year primary ischemic outcome at the expense of a 150% increase in bleeding events in elderly patients 1 to 3 years after a prior MI [19]. The use of ticagrelor and aspirin in patients with diabetes mellitus and stable CAD was shown to have a modest benefit on the primary ischemic outcome at the expense of increased bleeding rates in the THEMIS (The Effect of Ticagrelor on Health Outcomes in Diabetes Mellitus Patients Intervention Study) trial. This benefit was not significant when stratified by elderly patients over 75 years of age [20]. An analysis of the RENAMI (REgistry of New Antiplatelets in patients with Myocardial Infarction) registry showed reduced fatal and non-fatal ischemic events by extending DAPT with prasugrel or ticagrelor beyond 12 months. However, this benefit was reduced in patients older than 75 years due to an increased risk of bleeding [21].

This systematic review and meta-analysis provides important insights on DAPT for elderly patients with ACS that may inform decisions in clinical practice. Elderly patients with ACS have an increased risk for bleeding events that can offset the ischemic benefit of DAPT. A risk assessment should be completed before initiating DAPT and new guideline recommendations indicate that bleeding risk should be the priority for informing decision making [22]. Individual risk assessments that account for quantitative and qualitative metrics are required since the causes of bleeding are variable and multifactorial within the elderly population [23]. The PRECISE-DAPT and PARIS risk scores have shown modest accuracy in predicting bleeding risk in elderly patients [24]. As drug-eluting stents continue to improve, the use of a shorter duration of DAPT offers a potential bleeding risk mitigation strategy for elderly patients [25, 26]. P2Y_12_ monotherapy versus DAPT is another potential strategy to reduce bleeding events in elderly patients with CAD currently being investigated [27, 28]. Genotype-guided P2Y_12_ inhibitor selection is another area of research that may provide clinical benefits to elderly patients [29].

The limitations of this systematic review and meta-analysis are influenced by the limitations of the included studies. All of the included studies are likely influenced by between-center variability and the lack of centralized independent assessment of procedural results and outcomes. Antiplatelet therapy regimens and follow-up time also differ in each study and limit the generalizability of the aggregate data. The statistical heterogeneity of the meta-analysis varied by outcome likely due to clinical and methodological diversity between studies.

## 5. Conclusion

This systematic review and meta-analysis suggests that DAPT with prasugrel or ticagrelor compared to clopidogrel is associated with a higher risk of bleeding events in elderly patients with acute coronary syndrome. There was no difference in the primary efficacy endpoints between the two treatment groups.

## Figures and Tables

**Table 1:** Baseline characteristics of the included studies.

Study Author Year	Trial	Sample size	Group	Gropu size	Mean/Median,Age,Years	Female	White race%	BMI,Kg/m^2^	Diabetesmellitus,%	Hypertension	Currentsmoker	PriorPCI,%	PriorCABG,%	Type ofACs,STEMI,%	Type of ACs, NSTEMI,%
Wiviott et al, 2007	TRITON-TIMI 38	13,608	Prasugrel	6813	61 (53–69)	25	92	28 (25–31)	23.0	64.0	NR	NR	8.0	26.0	74.0
			clopidogrel	6795	61 (53–70)	27	93	28 (25–31)	23.0	64.0	NR	NR	7.0	26.0	74.0

Wallentin et al.,2009	PLATO	18,624	Ticagrelor	9333	62.0	28.4	91.8	27 (13–62)	24.9	65.8	36.0	13.6	5.7	35.7	42.9
			clopidogrel	9292	62.0	28.3	91.1	27 (13–70)	25.1	65.1	35.7	13.1	6.2	35.8	42.5

Roe et al.,2013	TRILOGY ACS	9,326	Prasugrel (Age > 75)	1043	80.0 (77.0–83.0)	49.9	NR	NR	34.9	87.5	7.4	20.7	17.3	NR	79.5
			Clopidogrel (Age > 75)	1040	79.0 (77.0–83.0)	51.1	NR	NR	35.1	87.4	8.2	18.4	15.4	NR	77.3

Wang et al, 2016		200	Ticagrelor	100	79 (76–85)	31	NR	NR	42	79	37	3	0	37	44
			clopidogrel	100	80 (74–86)	34	NR	NR	39	82	41	65	0	32	47

Savonitto et al.,2018	ELDERLY ACS II	1443	Prasugrel	713	80 (77–84)	41.0	NR	26 (24–28)	30.0	78.0	9.0	20.0	8.0	42.0	48.0
			clopidogrel	730	80 (77–84)	39.0	NR	26 (24–28)	28.0	78.0	9.0	16.0	10.0	41.0	47.0

Schmucker et al.,2019	BREMEN-STEMI	1087	Ticagrelor	535	80.9 ± 4.7	49.9	NR	26.1 ± 4.1	20.6	NR	9.6	11.7	3.2	67.5	NR
			clopidogrel	552	80.9 ± 4.6	51.1	NR	25.9 ± 4.7	24.1	NR	14.3	10.2	2.9	60.2	NR

Szummer et al.,2020	SWEDEHEART	14,005	Ticagrelor (After IPTW)	5,607	85.0 ± 3.9	51.8	NR	NR	22.2	69.1	5.6	13.3	9.0	30.9	NR
			Clopidogrel (After IPTW)	8,421	84.0 ± 3.9	51.6	NR	NR	22.4	68.6	5.6	13.3	9.0	31.4	NR

Gimbel et al.,2020	POPular AGE	1002	Ticagrelor or prasugrel	502	77 (73–82)	35	NR	26.9 ± 4.2	30	73	13	24	17	NR	86
			clopidogrel	500	77 (73–81)	37	NR	26.7 ± 4.0	29	73	14	20	17	NR	86

Data are median (IQR), mean ± standard deviation, or percentages as indicated. BMI = body mass index, PCI = percutaneous coronary intervention, CABG = coronary artery bypass graft, ACS = acute coronary syndrome, STEMI = ST elevation myocardial infarction, NSTEMI = non-ST elevation myocardial infarction, and IPTW = Inverse Probability Treatment Weighting.

## Data Availability

The data used are included within the article.
